# A Link between Species Abundance and Plant Strategies for Semi-Natural Dry Grasslands

**DOI:** 10.3390/plants13162260

**Published:** 2024-08-14

**Authors:** Sonja Škornik, Nataša Pipenbaher

**Affiliations:** Department of Biology, Faculty of Natural Sciences and Mathematics, University of Maribor, Koroška 160, 2000 Maribor, Slovenia; natasa.pipenbaher@um.si

**Keywords:** species coexistence, biodiversity, community assembly, species dominance, subordinate species, *Festuco-Brometea*, plant functional traits, community structure, Goričko, NE Slovenia

## Abstract

Due of the potential of species to determine ecosystem properties, it is important to understand how species abundance influences community assembly. Using vegetation surveys on 35 dry grasslands in north-east Slovenia, we defined dominant (8) and subordinate (61) plant species. They were compared on 14 traits to test for differences in community-weighted mean (CWM) and functional diversity (FD). We found that dominants and subordinates differed strongly in their functional traits. Dominants showed higher leaf dry matter content and a more pronounced stress tolerance strategy and were all clonal with a large proportion of species with rhizomes and a rich bud bank, while other species showed a higher specific leaf area, a longer flowering period and more ruderals. For most traits, FD was higher in subordinates. Our results suggest that dominants drive community structure by limited susceptibility to non-competitive processes. Dominants may have positive effects on subordinates by mitigating environmental stressors. Subordinates are able to assemble together by being dissimilar and use different fine-scale niches that are engineered and homogenised by dominants. Our results show that there are fundamental differences in the relative importance of ecological processes between dominant and subordinate plants in species-rich grasslands, which is also important for their conservational management.

## 1. Introduction

Plant diversity is an important driver of ecosystem functioning [1] and contributes significantly to the provision of ES [2]. Despite conflicting views on the relationship between species richness and ecosystem functioning, many scientists suggest that only a few species are needed to sustain most processes and functions [3,4,5]. Both theory and experimental evidence [6,7,8,9] suggest that ecosystem properties should be largely determined by the characteristics of dominant species. When ecosystems degrade to the point where formerly dominant species disappear or become rare, it is often possible to detect a decline in ecosystem functions and ES [10,11]. Therefore, the identification of patterns related to the maintenance of a certain community species composition and, in particular, dominant species should be mandatory when studying ecosystem functioning [12,13]. There are several studies focussing on processes related to relatively dominant species [7,8,9,14,15,16,17]. As reported by Tilman [18], the abundance of a species is proportional to the amount of the habitat that has the environmental conditions that meet the species’ requirements. Previous studies have shown [14] that there are two primary routes to dominance: superior access of dominant species to limiting resources through competition or limited susceptibility of dominant species to non-competitive processes that are highly restrictive for other species. However, there are fundamental differences in the relative importance of ecological processes between dominant and non-dominant plants [19]. For example, when dominant plants are constrained by the environment (strong habitat filtering), they may not deplete available resources, but instead may mitigate environmental stressors that normally constrain non-dominant plants [16]. Therefore, to understand dominance, the relationship between the mechanisms that cause the abundance of the dominant species and the mechanisms that limit the abundance of the other species must be determined, and not only the influence of the dominant species on the limiting resources [14].

To explain why some species are more abundant than others in a given habitat, it is helpful to compare the plant functional traits (PFTs) of the species present in a habitat, as PFTs determine a species’ response to resource availability, competition from neighbouring plants and herbivory [20]. Strong habitat filtering should result in traits being more similar than expected by randomness (clustering or underdispersion), while limiting similarity (niche differentiation) should generate overdispersion [21].

If the immediate influence of vegetation on ecosystem properties is primarily determined by the traits of dominant species [19], it is necessary to consider what additional effects other (rare or subordinate) species might have in plant communities. According to Tilman [18], less common species are better adapted to the less common environmental conditions in a habitat and probably possess different functional traits than the common species [22]. Indeed, previous studies have shown that rare and subordinate species increase species richness [22] and the functional diversity of communities [23]. Moreover, several studies [19,22] have emphasised the role of overall diversity in ensuring the stability and functioning of ecosystems [12,13,24,25].

Semi-natural grasslands (meadows and pastures) are a particularly suitable example for analysing this specific topic. They represent an important land-use type in Europe that plays a fundamental role in regulating ES, e.g., by reducing erosion by supporting slope stability, regulating the water balance and purifying water from fertilisers and pesticides [26]. Extensively managed semi-natural grasslands offer a high level of biodiversity and harbour many rare and endangered species from different taxonomic groups [27,28]. Due to their importance, they are listed in Annex I of the EU Habitat Directive (CE 43/92) as priority habitats, i.e., as “natural habitat types in danger of disappearance” [22].

The main objective of this work was to investigate why some species are more abundant than others in a semi-natural dry grassland community in north-eastern Slovenia, to better understand the mechanisms of community assembly that influence dominant and subordinate species. To achieve this goal, we analysed the plant traits of the grassland species. We addressed the following questions: (i) How do the dominant and subordinate plant species occurring in the studied grassland community differ in their plant functional traits (hereafter PFTs)? (ii) What are the differences in the ecological strategies of plants between dominant and subordinate plant species? While the species diversity and composition of grasslands is primarily the result of management, changes in land use can alter this structure and affect community stability and ecosystem functioning [23]. Acquiring knowledge on this topic is of great importance for the conservation and restoration of this highly diverse (both taxonomic and functional) plant community and the ecosystem functions it provides, as well as for the development of appropriate conservation measures.

## 2. Results

Across 35 relevés of studied dry grasslands (association *Hypochoerido-Festucetum rupicolae*), we identified 101 vascular plant species. The mean number of species per relevé was 38 ± 6. From 101 plant species, 69 species reached at least 1% cover in at least one relevé. Eight (8) species reached more than 25% cover in at least one relevé and were selected as dominants (Appendix A): *Anthoxanthum odoratum*, *Briza media*, *Centaurea jacea*, *Dianthus carthusianorum*, *Festuca rubra*, *Festuca rupicola*, *Hieracium bauhinii* and *Peucedanum oreoselinum*. Of these, only *Briza media* and *Festuca rupicola* reached 60% cover in at least one relevé. The dominant species belong to the following families: *Poaceae* (four dominants), *Compositae* (two), *Apiaceae* (one) and *Caryophyllaceae* (one). Sixty-one (61) species were selected as subordinates (list of species in Appendix A).

**Figure 1:**
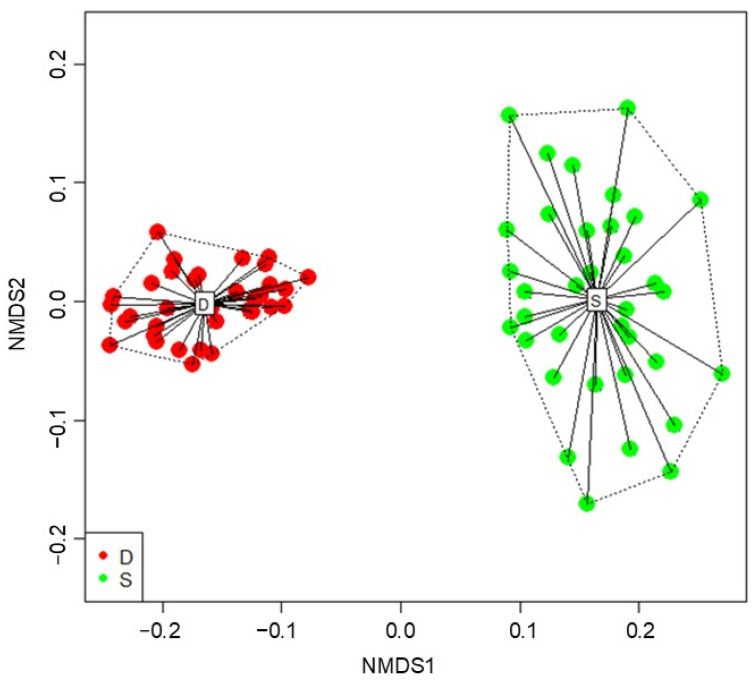
A nonmetric multidimensional scaling (NMDS) plot showing differences in CWM within and between dominant (D) and subordinate (S) plant species. A Euclidian distance similarity matrix was calculated based on the 35 vegetation relevés and 35 plant functional traits (CWM1) for dominants (D) and on the 35 vegetation relevés and 35 plant functional traits (CWM2) for subordinates (S). Relevés with dominants and subordinates are illustrated by different colours: red circles—dominant (D) plant species; green circles—subordinate (S) plant species.

The PFT differences between dominants and subordinates were first analysed using the PERMANOVA analysis, which showed significant differences in the community-weighted means (CWM) (*p* < α, α = 0.05) between the observed groups. The NMDS (Euclidean distance) plot (Figure 1) showed not only the strong differences in the composition of functional traits between dominant and subordinate plant species, but also that there were higher differences in CWM values within the subordinate species compared to CWM values within the dominant species. This was also confirmed by the significant (*p* < α, α = 0.05) beta-dispersion test.

**Figure 2:**
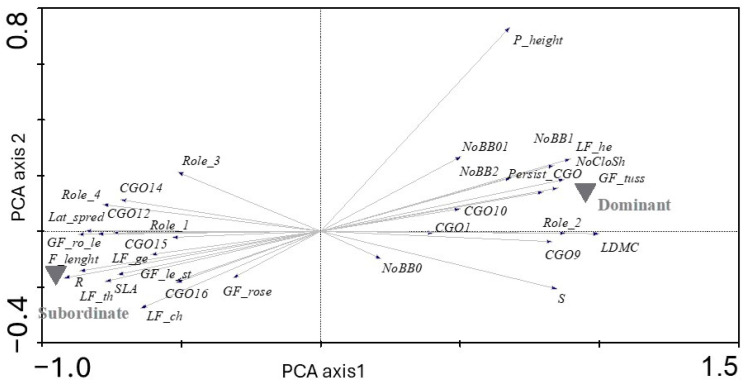
PCA ordination diagram of CWM matrix with 35 relevés × 32 plant functional traits. Only traits (*n* = 32) with significant differences in their CWM values between dominant (*n* = 8) and subordinate (*n* = 61) plant species (Student’s *t*-test) are shown. Eigenvalues: axis passively projected groups: dominant species, subordinate species. Abbreviations of plant traits are explained in Table 1.

In the second step, we tried to identify the predominant plant traits for dominant and subordinate plant species with PCA (Figure 2). Only PFTs (*n* = 32) with significant differences between the two species groups were plotted in the ordination plot, where the position of passively projected classes was also indicated. Dominant and subordinate species were again clearly separated, indicating that both groups differ in composition of community traits. Comparison with Student’s *t*-test revealed significant differences in plant life and growth form, with dominants showing a significantly higher CWM for hemicryptophytes (LF_he) and tussock plants (e.g., grasses) (GF_tuss), while the CWMs for all other life forms (chamaephytes (LF_ch), geophytes (LF_ge) and therophytes (LF_th)) and growth forms (rosette (GF_rose), leafy stem (GF_le st), rosette and leafy stem (GF_ro le)) were significantly higher for subordinates.

Dominant plant species had significantly higher CWM values for plant height (P_height) and leaf dry matter content (LDMC), with more species classified as stress tolerators (S), but lower CWM values for ruderals (R) and specific leaf area (SLA).

The two groups of coexisting species differed in most clonal plant traits (Appendix B). Compared to the subordinate plants, the dominant species had significantly higher CWM values for stolons (CGO1) and epigeogenous (CGO9) and hypogeogenous rhizome (CGO10). Community mean values of clonal growth traits also showed that dominants had more persistent connections between ramets and a higher multiplication rate (number of clonal offspring per mother plant, NoCloSh), as well as a higher proportion of species for which clonal organs are necessary to complete the life cycle (Role_2) (Appendix B). In contrast, among the subordinate species, there were more species with belowground stems (CGO12), root tubers (CGO16), root splitters (CGO14) and roots with adventitious buds (CGO16). Their lateral spread is more intense (Lat_spread), but the connections between ramets are less persistent (Persist_CGO). Among the subordinates, there are more species than among the dominant ones for which CGO is not necessary to complete the life cycle and whose role is therefore considered additive (Role_1) or which play a role in regeneration after injury (regenerative role, Role_3) or which have no role (Role_4). For the bud bank traits, the highest number of plant bud banks was found in all layers (NoBB1, NoBB2, NoBB0, NoBB01) in the dominant species. There were no significant differences between dominants and subordinates for the traits of flowering start, bulbs as clonal organs (CGO13) and competitors (C) (Appendix B). The analysis of the flower phenology showed that the group of dominants had a longer flowering period (F_length).

In the third step, we looked for differences between the groups in functional diversity (FD) (Appendix B). The analysis showed that dominants and subordinates differed significantly in FD for most traits and/or trait groups (15 out of 18), and for all traits, we found greater FD for subordinates compared to dominant plant species. There were no significant differences in FD for plant height, specific leaf area (SLA) and competitors (C) between the two groups of plant species (*p* < 0.05) (Appendix B).

**Figure 3:**
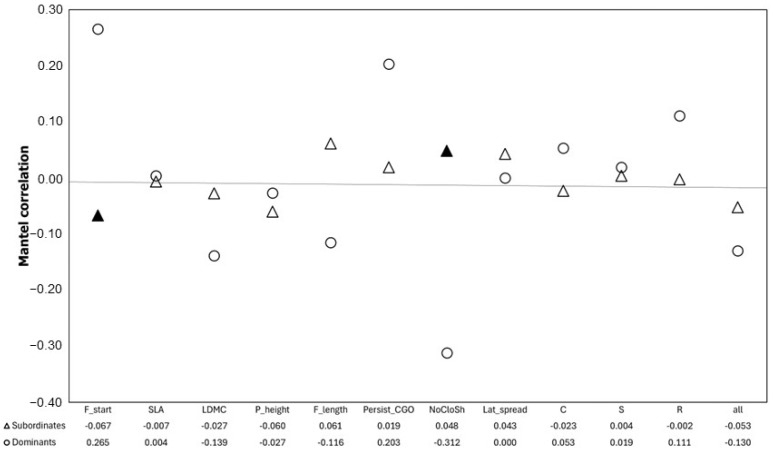
Divergence and convergence patterns of dominant and subordinate plant species. Results of Mantel correlations between species dissimilarity and species co-occurrence matrices. Field symbols indicate significant (*p* < 0.05) results. The presented results refer only to continues traits. Abbreviations of plant traits are explained in Table 1.

Finally, we presented divergence and convergence patterns between coexisting dominant and subordinate plant species for all continuous traits (Figure 3). We detected both divergence and convergence; however, we found a significant deviation from random expectations only for subordinate species and only for a small subset of traits. Significant trait convergence (negative correlation between species co-occurrence and species trait dissimilarity) was found for the flowering start (F_start) and significant trait divergence for the number of clonal offspring per mother plant (NoCloSh) (Figure 3).

## 3. Discussion

**Figure 4:**
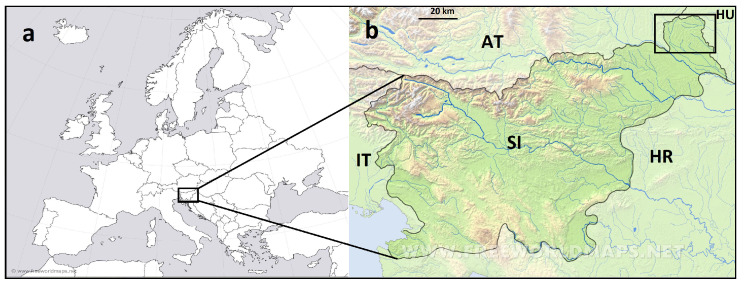
Geographic location of (**a**) Slovenia in the map of Europe, and (**b**) the study area (black square) in the map of Slovenia. Source: www.freeworldmaps.net (accessed on 3 August 2024) 2005–2021.

The species identified in the studied plant community represent typical species of extensively used dry grassland vegetation in the area of the Goričko Landscape Park (GLP, Figure 4) [34]. As the name suggests, grasslands are often dominated by grass species (Poaceae) [16], and here, we report on four dominant grasses, including the species *Festuca rupicola* with the highest cover values recorded in the studied grasslands. However, no species reached a cover of more than 80% in the relevés, which means that there are no dominant species that could be considered as monopolist [35,36]. Thus, the coexistence of numerous species in this plant community is possible.

We found that the dominant and subordinate species in a dry grassland community differ greatly in their functional traits. Our results are consistent with other findings [16,25,37] that indicate fundamental differences in the relative importance of ecological processes between dominant and non-dominant plants. We observed that there are several traits that contribute to the dominance of a species in these habitats. Since graminoids, which made up a significant proportion (50%) of dominant species, tend to have very similar structures (shoots and roots) [18], dominant species were more similar to each other, while subordinate species showed greater variation in traits. Dominant species had higher LDMC and lower SLA values than others. Species characterised by lower SLA have slower tissue turnover with a longer leaf life span [38] and a large investment in high-density tissues for which LDMC is a good estimator [39]. Species with higher LDMCs are better at resource conservation [40] and tend to have physically stronger leaves and are therefore likely to be better protected against abiotic (e.g., wind or hail) and biotic mechanical damage (e.g., herbivory) [41,42,43]. The observed results suggest that traits associated with more stressful habitat conditions (habitat filtering) are associated with dominance. This was also supported by our finding that species with an S-strategy were positively associated with dominance. Dominant species tended to have more resource-deficient strategies than subordinate species, which may indicate that species were exposed to a scarcity of mineral nutrient supply in the grassland community studied [44]. As previously documented in other studies [34,45], grasslands of the *Hypochoerido-Festucetum rupicolae* association are low-productivity habitats with low soil fertility, which has been associated with acidic soils [34] and vegetation management [46]. They are of semi-natural origin, i.e., they have developed on former forest land and are maintained by traditional (or similar) management practises, mowing once or twice a year and with no or very limited additional fertilisation [47]. In our study, we found that clonal perennial herbs dominate with a large proportion of species with epigeogeonous and hypogeogenous rhizomes and a rich bud bank in all layers. In general, rhizomes are very common in grassland species [48]. This investment in belowground structures by clonal plants has several advantages, such as overcoming the negative effects of environmental heterogeneity through ramet integration [49,50], faster space utilisation (lateral spread) and vegetative propagation [43,51]. In addition, clonal organs bear buds and store carbohydrates that can enable successful recovery after disturbance [52,53].

The differences in functional diversity between dominants and subordinates are related to the different number of available niches along the ecological gradients [54]. According to our results, diversity for most traits was higher in the subordinate species. In these species-rich dry grasslands, the functional variation of the traits considered is evidence of an equalisation of fitness of the non-dominant (subordinate) species that allows them to coexist regardless of their trait differences [55]. This equalisation is consistent with the hypothesis that dominant species may have positive effects on non-dominant species by mitigating environmental stressors that affect them, rather than dominant species depleting resources and increasing environmental stress for non-dominant species [16]. Furthermore, our results showed that species with a ruderal (R) strategy were associated with non-dominance. According to Grime [56], functional diversity is more pronounced under moderately disturbed conditions—such as in traditionally grazed or mowed grasslands [57] where biomass removal promote species with low competitive ability [58]. The low relative proportion of ruderals (R) in both dominant and subordinate species also indicates a moderate influence of disturbance in the studied semi-natural grassland community, which can be attributed to the continuation of the low-intensity management regime [46]. Although disturbance is known to be the strongest force that creates and maintains the coexistence of functionally different species [56,59], this decreases at high disturbance frequencies [60]. Favourable conditions for ruderal species may occur on grassland exposed to land-use intensification or to the long-term effects of local disturbances, such as uprooting of plants and trampling by large and small mammals, including humans, while on arable land, the most severe damage occurs on trackways [59]. Several studies have shown that increasing disturbance (i.e., intensification of land use) can severely affect the structure and function of grassland plant communities [23,59]. As the native flora often shows that the rate at which it is reduced or eliminated by disturbance is much faster than the rate at which most native plants spread, recovery of disturbed grasslands may be circumvented by species that are less restricted by immigration into the current landscape, i.e., exotic species [14] and ruderal/weed species that are also very common in the surrounding agricultural areas of the GLP studied [61].

When analysing divergence and convergence between plant species within each of the two groups, we found significant deviation from random expectations only for subordinate species and only for a small proportion of traits. Significant trait convergence (negative correlation between species co-occurrence and species trait dissimilarity) was found for flowering start, suggesting that the co-occurrence of subordinate species within 25 m^2^ plots (relevés) was greater between co-flowering species. Flowering phenology is a crucial element of plant ecology and an important component of community assembly [22,62,63]. It influences the presence or absence of species in a habitat and also their relative abundance [62]. Precise phenological timing is certainly linked to climate [22,64]. Most temperate species flower in response to temperature, which determines the beginning and end of the growing season [65]. In our case, the similar flowering pattern is probably an example of adaptation to the arid soil conditions in this habitat. The dry grasslands in this study occur on sites with shallow sandy soils on sunny, south- and south-west-facing slopes, where drought often restricts plant growth in summer [35]. In contrast, we found that the number of clonal offspring per mother plant was, on average, divergent between subordinate species in the dry grasslands studied. Our result supports the observed differences between dominant and subordinate species when comparing functional diversity and is consistent with the prediction of Stubbs and Wilson [66] that subordinate species are able to assemble together by being dissimilar and thus utilising different fine-scale niches [66,67], under relatively homogeneous environmental conditions [64] established and homogenised by dominants [16,68]. Subordinate species are often considered as those that exploit marginal conditions or barely survive with the dominant species. However, subordinate species may also benefit from dominant species due to more stable microclimatic conditions, reduced herbivory, pathogens or other negative mechanisms [69,70].

Although our study is limited in terms of the number of vegetation samples (relevés), it covers the entire study area of the Goričko Landscape Park and includes most still-existing dry grasslands, thus aiming at understanding the mechanisms of community assembly affecting coexisting species. Our results suggest that dominant species are more likely to be influenced by the environment than subordinate species, while subordinates are facilitated by dominant species in dry grasslands. This finding should be considered when designing management measures for the conservation of dry grasslands, as it is crucial to maintain environmental conditions that allow the abundance of dominant species and thus a high level of floristic and functional diversity.

## 4. Materials and Methods

### 4.1. Study Area and Field Methods

The study area is located in Goričko Landscape Park (hereafter GLP) in the north-eastern part of Slovenia, at approximately 46° N, 16° E (Figure 4). With an area of 463.5 km^2^, GLP is part of the Trilateral Park: Goričko (Slovenia), Raab (Austria) and Őrség (Hungary) [71]. The GLP has been a Natura 2000 area since 2002 (site name: Goričko, code: SI5000009), with the aim of preserving traditional and extensive small-scale farming [71,72]. The climate is moderate continental or sub-Pannonic with dry winters. The average annual rainfall is between 500 and 600 mm [73]. The driest months are February and March, while most of the precipitation falls in July [73]. The average annual temperature is between 9 and 10 °C [73]. It is a hilly region (altitude between 300 and 350 m above sea level), with acidic, non-carbonate bedrock. The soil consists mainly of Ranker and Pseudogley [34]. The GLP is a mixture of forest and open areas. Half of the area is agricultural land with a mosaic of fields, semi-natural grasslands, orchards, vineyards, hedges or small groups of trees [71].

Dry grasslands in the GLP are extensively used, mowed once or twice a year, unfertilized and without additional seeding of grass and/or plant species [34]. Their main environmental characteristic is a highly acidic soil (pH approx. 5), which is low in nutrients and has low annual biomass production [61]. In the last decade, the area of extensive grasslands in the eastern part of the GLP has decreased by over 30%, which is due to the intensification of agricultural practises or abandonment [46]. The semi-natural dry grasslands studied are covered by the EU Habitat Directive and are classified as semi-natural dry grasslands and scrubland facies on calcareous substrates (Festuco-Brometalia) (* important orchid sites), code 6210(*).

In this study, we used a dataset of 35 grassland samples (phytosociological relevés) from our own database (relevés collected by the authors of this study). The study area of the Goričko Natural Park (Figure 4) was systematically searched during the field survey conducted at the peak of vegetative growth in June 2013 and 2015. We identified all grassland patches corresponding to habitat type 6210, semi-natural dry grasslands and scrubland facies on calcareous substrates (Festuco-Brometalia). This vegetation has already been described [34] using the phytosociological approach of Braun-Blanquet [74]. Based on their floristic composition and the occurrence of characteristic species, they were assigned to the association *Hypochoerido-Festucetum rupicolae* Steinbuch 1995 (order *Brometalia erecti*, class *Festuco-Brometea*) [34,75]. We collected data on vegetation composition of all dry grassland stands representing the association *Hypochoerido-Festucetum rupicolae* in a favourable conservation status (i.e., typical physiognomy and species composition). The species composition of grassland areas was recorded in plots of 5 m × 5 m each. In each 25 m^2^ plot, the vascular plants were recorded using a seven-point cover–abundance scale (*r*, +, 1, 2, 3, 4, 5) according to the Braun-Blanquet method [74]. The taxonomic classification was carried out according to Martinčič et al. [29].

The cover–abundance data were used to select those species that dominate in the analysed plant community. Before the further steps, the values of the alphanumeric Braun-Blanquet scale were converted into cover % values as suggested by Van der Maarel [76] (r = (0), + = 0.1, 1 = 5.0, 2 = 17.5, 3 = 37.5, 4 = 62.5, 5 = 87.5). For the selection of species, we followed the modified protocol described by Prach and Pyšek [35]. The species occurring in the relevés (only those that achieved a cover of at least 1% in at least one relevé, value 1 according to the Braun-Blanquet scale) were categorised into two groups: (1) dominant species were those whose cover was above 25% in at least one relevé (values 3, 4 and 5 according to the Braun-Blanquet scale); (b) subordinate species were those that did not fulfil this criterion.

### 4.2. Selected Plant Functional Traits

To characterise dominant and subordinate plant species, we obtained PFT data from the literature [29], from our own database (protocol standardised by Hodgson [33]) and from the existing trait databases CLO-PLA3 (a database of clonal growth of plants from Central Europe) [31,32] and LEDA [30]. The species were characterised using thirteen basic traits and one composite trait (CSR strategy) [44]. The list of PFTs with the description of the classes in the matrix, the units and the sources of information is presented in Table 1. The following traits were selected: life form, growth form, plant height, specific leaf area (SLA), leaf dry matter content (LDMC), flowering start and flowering length, type of clonal growth organ (CGO), persistence of connection in CGO, number of clonal offspring shoots, lateral spread distance by clonal growth, role of CGO in life-history of a plant, size of plant bud bank (including root buds) and CSR strategy. The categorical PFTs (life form, growth form, type of CGO, role of CGO and plant bud bank) were transformed into binary variables, where 1 represents the occurrence of the trait. In this way, the number of traits in the matrix increased from 14 to 35.

The CSR strategy scheme provides a system for classifying herbaceous plants according to strategies adapted to competition (C), abiotic stress (S) and disturbance (R) [60]. There are methods to classify species as C, S or R strategy based on simple PFT [33]: plant height (cm), LDMC (mg/g), flowering length, flowering start, lateral spread, leaf dry mass (g) and SLA (mm^2^/mg). To determine the CSR strategy, we used the spreadsheets from [33], which were made available for this purpose at http://www.ex.ac.uk/~rh203/allocatingcsr.html (accessed on 21 February 2024). For the purpose of multivariate analyses, we chose to express CSR strategy as three continuous variables (C, S, R) to reflect the degree of species adaptation to each of the CSR axes.

### 4.3. Functional Trait Indices

To assess the composition of functional traits, we combined the species by relevé matrix with the species by trait matrix. First, we calculated the community-weighted mean trait values (CWM) for each relevé as the average of the trait values weighted by the relative abundance of each species [54,77,78,79,80]. The metric is simply calculated as follows:

$${\mathrm{CWM}}_{jk}=\overset{S}{\underset{i=1}{\sum }}p_{ik}\times x_{ij}$$

where CWM*_jk_* is the community-weighted mean value of trait *j* at site *k*, *p_ik_* is the relative abundance of species *i* (*i* = 1, 2, …, *S*) at site *k* and *x*_ij_ is the value of trait *j* for species *i* [81]. This operation results in two matrices: The first matrix was formed by combining dominant species (*n* = 8) by relevés (*n* = 35) with dominant species (*n* = 8) by traits (*n* = 35); this operation results in matrix CWM1 of 35 traits by 35 relevés. The second matrix was formed by combining subordinate species (*n* = 61) by relevés (*n* = 35) with the matrix of subordinate species (*n* = 61) by traits (*n* = 35). This operation results in matrix CWM2 with 35 traits by 35 relevés.

We also calculated the functional diversity index (hereafter FD) proposed by Lepš et al. [78] to measure the functional diversity index of individual traits using Rao (1982) quadratic diversity. The metric is calculated as follows:

$$\mathrm{FD}=\overset{S}{\underset{ij}{\sum }}d_{ij}\times p_{i}\times p_{j}$$

where the proportion of the *i*-th species in a community is *p_i_* and the dissimilarity of species *i* and *j* is *d_ij_*. *S* is the number of species in the community. Calculations were performed using an Excel macro 97 [78]. This operation results in a matrix of 18 FD indices for each trait by 35 relevés (matrix FD). Calculations were performed separately for dominant and subordinate species.

### 4.4. Data Analysis

To assess the proportional differences in the community-weighted mean (CWM) with and between dominant and subordinate plant species, a PERMANOVA analysis was performed using the packages VEGAN [82] and LATTICE [83] in the R statistical environment 4.2.0 [84]. We worked with the Euclidian distance parameter and 999 permutations. To visualise the results, an NMDS approach was performed with the “vegan” R package [82,85]. The betadisper function and the corresponding ANOVA were used to test for differences or similarities in data dispersion within and between the dominant and subordinate plant species.

To test for differences in CWM and FD indices between dominant and subordinate plant species, we analysed the data using Student’s *t*-test for independent samples (R Development Core Team, 2009) (Appendix B). The CWM indices were further analysed using Principal Component Analysis (PCA) [86]. Only traits with significant differences in their CWM values between dominant and subordinate plant species (Student’s *t*-test) were used for PCA. The gradient length for the first PCA axis of ordination was less than three, indicating that linear ordination methods are suitable for the analysis. The ordination method (PCA) and visualisation of its results were performed using the programmes Canoco and CanoDraw [87].

In addition, a series of Mantel tests [88] were used to examine the associations between the V-coefficient matrices and the trait dissimilarity matrices for divergence and convergence patterns between coexisting dominant and subordinate plant species for all continuous traits. The analysis was performed with the VEGAN package [80] in the R statistical environment (R Development Core Team 2021).

## 5. Conclusions and Conservation Implications

Although numerical abundance suggests competitive dominance, our analysis of the structure of a semi-natural dry grassland community in relation to dominant and subordinate species revealed that dominant species drive community structure through limited susceptibility to non-competitive processes that are restrictive to other (subordinate) species [44,89]. Our results are consistent with the hypothesis that dominant species (which are constrained by the environment) may have a stronger positive effect on some subordinate species by mitigating environmental stressors that normally constrain subordinate species. Therefore, the maintenance of the traditional management system should be included in the management plans for conservation or restoration, as it is crucial to maintain the typical abiotic and biotic conditions that guarantee the preservation of the typical floristic composition and the high level of floristic and functional diversity.

## Figures and Tables

**Table 1:** Plant functional traits (*n* = 14) recorded for 69 vascular plant species of semi-natural dry grasslands. Scale of measurement was originally categorical (cat) or circular (circ) or continuous (cont).

Plant Functional Traits (PFTs)		Abbreviation and Description	Data Source
Life form	cat	LF_ch—chamaephytes; LF_ge—geophytes; LF_he—hemicryptophytes; LF_th—therophytes;	[29]
Growth form	cat	GF_tuss—tussocks; GF_rose—rosette; GF_le_st—leafy stem; GF_ro_le—rosette and leafy stem;	[29]
Plant height	cont	P_height (cm)	Own measurements
Specific leaf area	cont	SLA (mm^2^/mg)	Own measurements; LEDA database [30]
Leaf dry matter content	cont	LDMC (mg/g)	Own measurements; LEDA database [30]
Flowering start	circ	F_start (°)	[29]
Flowering length	cont	F_length (months)	[29]
Type of clonal growth organ	cat	CGO1—stolon CGO9—epigeogenous rhizome CGO10—hypogeogenous rhizome CGO12—belowground stem tuber CGO13—bulb CGO14—root splitters CGO15—root with adventitious buds CGO16—root tuber	CLO-PLA3 database [31,32]
Persistence of connection in CGO	cont	Persist_CGO (year)	CLO-PLA3 database [31,32]
Number of clonal offspring shoots	cont	NoCloSh	CLO-PLA3 database [31,32]
Lateral spreading distance by clonal growth	cont	Lat_spread (m)	CLO-PLA3 database [31,32]
Role of clonal growth organs	cat	Role_1—additive Role_2—necessary Role_3—regenerative Role_4—none	CLO-PLA3 database [31,32]
Bud bank of the plant	cat	NoBB1—bud bank on the plant higher than 10 cm NoBB2—bud bank on the plant 10 to 0 cm NoBB0—bud bank at the soil surface NoBB01—bud bank at a depth of 0 to 10 cm	CLO-PLA3 database [31,32]
CSR strategy	cont	C—competitors S—stress tolerators R—ruderals	Own measurements of PFT for CSR determination: plant height, LDMC, flowering length, flowering start, lateral spread, leaf dry mass and SLA (protocol by Hodgson et al. [33])

## Data Availability

Most data are contained within the article. The PFT data presented in this study are available on request from the corresponding author.

## Appendix A

**Table A1:** List of dominant (8) and other (61) plant species recorded in 35 vegetation stands (relevés) of semi-natural dry grasslands. Only species with >1% cover in at least one relevé were included. Species are arranged according to the frequencies (in percentage) of species occurrence in the relevés. The highest and lowest cover–abundance values (according to Škornik [34]) are also shown as superscripts. Legend: **^d^** dominants.

Species Name	Fr (%)
*Festuca rupicola * ^d^	100 ^1−4^
*Luzula campestris*	97 ^+2^
*Briza media * ^d^	91 ^+4^
*Ranunculus bulbosus*	91 ^+2^
*Leontodon hispidus* subsp. *hastilis*	86 ^+2^
*Hieracium bauhinia * ^d^	83 ^+3^
*Anthoxanthum odoratum * ^d^	83 ^+3^
*Dianthus deltoides*	83 ^+2^
*Achillea millefolium*	80 ^+1^
*Lotus corniculatus*	80 ^+1^
*Trifolium pratense*	80 ^+2^
*Centaurea jacea * ^d^	77 ^+3^
*Thymus pulegioides*	77 ^+2^
*Festuca rubra * ^d^	74 ^+3^
*Dianthus armeria*	74 ^+1^
*Plantago lanceolata*	71 ^+2^
*Hieracium pilosella*	69 ^+2^
*Leucanthemum vulgare agg.*	69 ^+1^
*Trisetum flavescens*	69 ^+1^
*Holcus lanatus*	66 ^+2^
*Pimpinella saxifraga*	66 ^+1^
*Carex caryophyllea*	63 ^+1^
*Galium verum*	60 ^+1^
*Tragopogon orientalis*	60 ^+1^
*Arrhenatherum elatius*	57 ^+1^
*Campanula patula*	57 ^+1^
*Euphorbia cyparissias*	57 ^+2^
*Danthonia decumbens*	54 ^+2^
*Peucedanum oreoselinum * ^d^	51 ^+3^
*Orchis morio*	51 ^+1^
*Polygala comosa*	51 ^+2^
*Agrostis tenuis*	49 ^+2^
*Moenchia mantica* subsp. *mantica*	49 ^+2^
*Rhinanthus minor*	49 ^+2^
*Avenochloa pubescens*	46 ^+2^
*Daucus carota*	46 ^+1^
*Hypochoeris radicata*	46 ^+1^
*Betonica officinalis*	43 ^+2^
*Rumex acetosella*	43 ^+1^
*Plantago media*	40 ^+1^
*Potentilla erecta*	40 ^+1^
*Prunella laciniata*	40 ^+1^
*Trifolium repens*	37 ^+1^
*Carex pallescens*	31 ^+1^
*Trifolium campestre*	31 ^+1^
*Silene nutans*	29 ^+1^
*Carlina acaulis*	26 ^+1^
*Cynosurus cristatus*	26 ^+1^
*Helianthemum ovatum*	26 ^+2^
*Polygala vulgaris*	26 ^+1^
*Sanguisorba officinalis*	26 ^+1^
*Sedum sexangulare*	26 ^+2^
*Linum catharticum*	23 ^+1^
*Knautia drymeia*	20 ^+1^
*Poa angustifolia*	20 ^+1^
*Potentilla reptans*	20 ^+1^
*Scabiosa triandra*	20 ^+1^
*Thesium linophyllon*	20 ^+2^
*Lychnis viscaria*	17 ^+2^
*Carlina vulgaris*	11 ^+1^
*Hieracium umbellatum*	11 ^+1^
*Dianthus carthusianorum * ^d^	9 ^13^
*Chamaecytisus supinus*	9 ^+1^
*Chamaespartium sagittale*	9 ^+2^
*Ornithogalum umbellatum*	9 ^+1^
*Sanguisorba minor*	9 ^+1^
*Filipendula vulgaris*	6 ^+1^
*Peucedanum cervaria*	6 ^12^
*Verbascum austriacum*	6 ^1^

## Appendix B

**Table A2:** Results of t-test for aggregated trait (CWM, community-weighted mean) values and functional trait diversity (FD) between dominant (*n* = 8) and subordinate plant species (*n* = 61). Student *t*-test: ** *p* < 0.01; *** *p* < 0.001; n.s. non significant.

Plant Functional Traits (PFTs)	Abbreviation of Specific PFT	CWM				FD			
		D	S	*p*	Sig.	D	S	*p*	Sig.
Life form	LF_ch	0	0.07	***	<0.001	0.000	0.317	***	<0.001
	LF_ge	0	0.03	***	<0.001				
	LF_he	1	0.81	***	<0.001				
	LF_te	0	0.09	***	<0.001				
Growth form	GF_tuss	0.66	0.25	***	<0.001	0.512	0.719	***	<0.001
	GF_rose	0.12	0.16	**	0.035				
	GF_le_st	0.21	0.35	***	<0.001				
	GF_ro_le	0.01	0.24	***	<0.001				
Plant height	P_height	42.97	32.75	***	<0.001	0.210	0.218	n.s.	0.465
Specific leaf area	SLA	17.69	21.36	***	<0.001	0.189	0.169	n.s.	0.065
Leaf dry matter content	LDMC	350.77	250.31	***	<0.001	0.195	0.214	***	0.001
Flowering start	F_start	140.75	140.15	n.s.	0.700	0.401	0.456	***	<0.001
Flowering length	F_length	2.95	3.91	***	<0.001				
Type of clonal plant organ	CGO1	0.12	0.06	***	<0.001	0.639	0.700	***	<0.001
	CGO9	0.46	0.22	***	<0.001				
	CGO10	0.31	0.22	***	<0.001				
	CGO12	0	0.04	***	<0.001				
	CGO13	0	0.00	n.s.	0.112				
	CGO14	0.10	0.28	***	<0.001				
	CGO15	0	0.06	***	<0.001				
	CGO16	0	0.02	***	<0.001				
Persistence of connection in CGO	Persist_CGO	3.69	3.04	***	<0.001	0.147	0.362	***	<0.001
Number of clonal offspring shoots	NoCloSh	2.31	1.87	***	<0.001	0.219	0.344	***	<0.001
Lateral spreading distance by clonal growth	Lat_spread	1	1.23	***	<0.001	0.000	0.245	***	<0.001
Role of clonal growth organs	Role_1	0	0.02	***	<0.001	0.139	0.471	***	<0.001
	Role_2	0.91	0.60	***	<0.001				
	Role_3	0	0.01	***	<0.001				
	Role_4	0.09	0.28	***	<0.001				
Bud bank of the plant	NoBB1	5	3.53	***	<0.001	0.000	0.143	***	<0.001
	NoBB2	5	4.35	***	<0.001	0.000	0.135	***	<0.001
	NoBB0	6.24	5.86	**	0.033	0.157	0.199	**	0.006
	NoBB01	12.55	11.10	***	<0.001	0.275	0.367	***	<0.001
CSR strategy	C	0.38	0.38	n.s.	0.996	0.219	0.224	n.s.	0.552
	S	0.41	0.30	***	<0.001	0.195	0.317	***	<0.001
	R	0.21	0.33	***	<0.001	0.117	0.228	***	<0.001
